# Comorbidity associated to *Ascaris suum* infection during pulmonary fibrosis exacerbates chronic lung and liver inflammation and dysfunction but not affect the parasite cycle in mice

**DOI:** 10.1371/journal.pntd.0007896

**Published:** 2019-11-25

**Authors:** Fabrício Marcus Silva Oliveira, Pablo Hemanoel da Paixão Matias, Lucas Kraemer, Ana Clara Gazzinelli-Guimarães, Flaviane Vieira Santos, Chiara Cássia Oliveira Amorim, Denise Silva Nogueira, Camila Simões Freitas, Marcelo Vidigal Caliari, Daniella Castanheira Bartholomeu, Lilian Lacerda Bueno, Remo Castro Russo, Ricardo Toshio Fujiwara

**Affiliations:** 1 Laboratory of Immunology and Genomics of Parasites, Department of Parasitology, Institute of Biological Sciences, Universidade Federal de Minas Gerais, Belo Horizonte, Brazil; 2 Laboratory of Pulmonary Immunology and Mechanics, Department of Physiology and Biophysics, Institute of Biological Sciences, Universidade Federal de Minas Gerais, Belo Horizonte, Brazil; 3 Laboratory of Protozooses, Department of General Pathology, Institute of Biological Sciences, Universidade Federal de Minas Gerais, Belo Horizonte, Brazil; McGill University, CANADA

## Abstract

Ascariasis is considered the most neglected tropical disease, and is a major problem for the public health system. However, idiopathic pulmonary fibrosis (IPF) is a result of chronic extracellular deposition of matrix in the pulmonary parenchyma, and thickening of the alveolar septa, which reduces alveolar gas exchange. Considering the high rates of ascariasis and pulmonary fibrosis, we believe that these two diseases may co-exist and possibly lead to comorbidities. We therefore investigated the mechanisms involved in comorbidity of *Ascaris suum* (*A*. *suum*) infection, which could interfere with the progression of pulmonary fibrosis. In addition, we evaluated whether a previous lung fibrosis could interfere with the pulmonary cycle of *A*. *suum* in mice. The most important findings related to comorbidity in which *A*. *suum* infection exacerbated pulmonary and liver injury, inflammation and dysfunction, but did not promote excessive fibrosis in mice during the investigated comorbidity period. Interestingly, we found that pulmonary fibrosis did not alter the parasite cycle that transmigrated preferentially through preserved but not fibrotic areas of the lungs. Collectively, our results demonstrate that *A*. *suum* infection leads to comorbidity, and contributes to the aggravation of pulmonary dysfunction during pulmonary fibrosis, which also leads to significant liver injury and inflammation, without changing the *A*. *suum* cycle in the lungs.

## Author summary

Ascariasis is considered a major problem for the public health system, which has an estimated 800 million infected people worldwide. It occurs in the United States, Africa, Asia, and Latin America, and is generally associated with poverty and precarious health conditions. Pulmonary fibrosis affects 14–63 people per 100,000 habitants/year, and is characterized by collagen deposition and alveolar wall thickening. The comorbidities caused by infections are commonly associated with pulmonary fibrosis exacerbations, poor prognosis, and high mortality. Despite the comorbidities caused by helminth infections, which display a pulmonary parasitic cycle such as that of *Ascaris*, there is no evidence relating to pulmonary fibrosis progression, possibly because *Ascariasis* is considered a neglected disease. We evaluated the role of *Ascaris* during pulmonary fibrosis. We considered two simple questions: (1) Whether *Ascaris* infection could protect or aggravate fibrosis (comorbidities) and (2) whether pulmonary fibrosis could change the cycle of *Ascaris* as a result of increased alveolar thickening, larvae retention, and the limitation of influx into airways. We answered both questions as follows: (1) *Ascaris* infection exacerbates pulmonary and liver injury and inflammation, but not fibrosis; and (2) Pulmonary fibrosis did not alter the course of *Ascaris* cycle in lungs during transmigration into airways, because *Ascaris* preferentially seeks and penetrates into the lung areas, which are thought to be preserved, but not into fibrotic areas.

## Introduction

Pulmonary fibrosis is a limiting and lethal disease caused by an atypical fibroblast activation that leads to a chronic and excessive collagen deposition in the lung parenchyma, which results in alveolar structure abnormalities with impact on pulmonary function; reducing alveolar gas diffusion, and also modifying the pulmonary mechanics by loss of lung elasticity and airway flow [1,2,3]. Idiopathic pulmonary fibrosis (IPF) is the most aggressive manifestation of pulmonary fibrosis, and characterized by abundant collagen within the alveolar wall, thereby thickening and collapsing the architecture [2,3]. However, it is not clear whether the intrinsic extracellular matrix disturbs without inflammation, or inflammatory events preceding excessive lung tissue scar [4,5]. The sites of alveolar epithelial injury are commonly replaced by foci of fibroblastic proliferation and differentiation in myofibroblasts, with exuberant deposition of extracellular matrix that causes destruction of alveolar-capillary units leading to loss of organ function [2,3]. In addition to alveolar-capillary damage, cytokines and growth factors such as transforming growth factor-β_1_ (TGF-β_1_), interleukin-1β (IL-1β), interleukin-8 (IL-8), and interleukin-17A (IL-17A) induces the fibroblasts proliferation and source of type I collagen [6,7,8], which also recruit and activates interstitial pulmonary leukocytes, thereby contributing to exuberant inflammation, and tissue injury [1,6]. In this context, the cytokines/chemokines source related to both fibroblasts and leukocytes activation has been suggested as possible inducer of pulmonary fibrosis, and also considered possible targets to treat IPF [1,2,9,10,11].

The etiology of IPF is unknown, and some patients exhibit distinct patterns of disease progression, which display a rapid and progressive clinical course related to periods of acute exacerbations during the IPF development [3,12]. There are reports that environmental pollution, smoking, viral infections, and genetic abnormalities may be involved in the development of acute exacerbations during pulmonary fibrosis [3]. The prevalence and impact of comorbidities on the clinical course of IPF is unclear [13]. However, IPF patients frequently experience various comorbidities, such as pulmonary infection, emphysema, pulmonary hypertension, lung cancer, gastroesophageal reflux, cardiovascular disease, diabetes mellitus, and obstructive sleep apnea [3,12,13,14]. Respiratory comorbidities, especially bacterial pneumonia, influence the mortality and prognosis of hospitalized patients with IPF [14]. Recent observational studies have reported associations between lung dysbiosis, mortality, and altered host defense gene expression, thus supporting the role of lung microbiota in IPF [15]; results demonstrate that lung microbiota contributes to the progression of IPF, which suggests that lung dysbiosis promotes alveolar inflammation and aberrant repair [15]. Among the most common pathogens found were *Streptococcus pneumoniae*, *Streptococcus aureus*, *Klebsiella pneumoniae*, and *Pseudomonas aeruginosa* [14]; although lung infections as comorbidity during IPF are generally associated with poor prognosis and high mortality. Despite the comorbidities caused by helminth infections, which have a pulmonary parasitic cycle such as *Ascaris* or *Strongyloides*, there is no evidence during the pulmonary fibrosis, because helminthiasis are considered neglected diseases [16,17].

Ascariasis is the most prevalent helminthic infection in the United States, and one of the most prevalent tropical diseases caused by *Ascaris* [16,18]. This high prevalence is associated with poverty and precarious health conditions of developing countries from Africa, Asia, and Latin America [17,19,20]. The parasitic life cycle of the *Ascaris* genus may be divided into two distinct phases after infection: (i) migration of parasitic larval stages through several tissues (intestinal mucosa, liver and lung/airways), namely larval ascariasis; and (ii) after larval ingestion, the establishment of adult worms in the lumen of the small intestine, which characterizes chronic infection. After the egg eclosion in host, they are hatched in the lumen of the small intestine by L3 larvae that penetrate the intestinal mucosa through the cecum, they gain access to the blood circulation and the portal space of the liver, and then they migrate through the liver, where the larvae gain circulation again, and finally migrate to the lungs (Looss Cycle). The larvae migrate from the alveolar capillaries through the pulmonary parenchyma, causing disruption of the alveolar walls. At this stage, larvae L3 change to L4 and ascend the bronchial tree, where they can be expectorated or swallowed; at this last stage, the larvae returns to the gastrointestinal tract, and reach sexual maturity to become male and female adult worms [21,22,23]. Related to this damage is the immune response induced by *A*. *suum* migration through the tissues during the parasitic cycle; the damage triggered a mixed local and systemic Th2 and Th17 immune response, in which triple consecutive infections with *A*. *suum* reduced parasite burden related to immune specialization and memory [24]. In fact, the host protection against *A*. *suum* infection depends on a humoral-dependent response, mostly by induction of IgG antibodies, which is associated with the production of IL-5 and IL-10 after vaccination in mice [24,25].

Recent studies have shown the direct role of helminth infections in association with other pulmonary diseases, such as virus infection and allergy. However, the immunomodulatory or immunopathogenic effects of *Ascaris* infection in association with other diseases are still controversial. Co-infection by *A*. *suum* and *Vaccinia virus* promotes increased pathology associated with the virus titer, by downmodulation of the frequency of circulating CD4+T cells producing IFN-γ, which are important to control the *Vaccinia virus* [26]. However, *A*. *suum* infection leads to immunomodulatory effects during natural infection, and it depends on the geographic distribution pattern as well as the population affected. It has been demonstrated that children from areas with high endemicity of infections with *Ascaris lumbricoides*, have low parasitic load of *Ascaris lumbricoides*, which becomes a protective factor against asthma manifestation and its symptoms. However, a high parasitic load is a risk factor, and contributes to high prevalence of severe asthma and its symptoms among these children [27]. Regarding the Th2 and Th17 triggered by the helminth infection, it is the same type of immune response found during the fibrogenesis process [28,29], but the role of both pulmonary fibrosis and ascariasis association remains unclear.

Therefore, we assumed that it is necessary to better understand the mechanisms involved in these two diseases separately and in comorbidity, to evaluate the development of lung lesions. In this context, the aim of our study was to investigate whether *A*. *suum* infection could modulate pulmonary fibrosis, and whether pulmonary fibrosis could change the course of the parasite cycle through the airways during *A*. *suum* infection in mice. Our results suggested that comorbidity during *A*. *suum* infection exacerbated pulmonary and liver injury, inflammation, and dysfunction, without modification in tissue fibrosis. Finally, we demonstrated that pulmonary fibrosis induced by bleomycin did not change the pulmonary cycle of *A*. *suum*, which still transmigrated preferentially into the airways through preserved and non-fibrotic areas of the lungs.

## Materials and methods

### Ethics statement

All experimental procedures described here were conducted according to the Brazilian Guidelines on Animal Work, and approved by the local Animal Ethics Committee (CEUA) of the Federal University of Minas Gerais, Protocol number 200/2018.

### Mice

For the experiments, we used female (*Mus musculus*) C57BL/6 mice, aged approximately 8 weeks, obtained from the Biotério Central of the Federal University of Minas Gerais. During the experimental period, the mice were fed with filtered water and commercial chow (Nuvilab Cr-1, Nuvital Nutrients, Brazil) ad libitum. They were kept in cages (50 × 60 × 22 cm) with sterile sawdust shavings, and the cages were changed and cleaned once a week. Mice were maintained at the Animal Facility of the Universidade Federal de Minas Gerais (UFMG) under controlled conditions of temperature (24 ± 1° C) on a 12 h light cycle (lights on at 07:00 h).

### Parasites

*A*. *suum* adult worms were collected from infected-pig intestines discarded by a slaughterhouse located in the city of Belo Horizonte, Minas Gerais, Brazil, during their normal activities with the carcasses. Adult worms were kept in PBS (0.4 M NaCl and 10 mM NaPO4), and taken to the Laboratory of Immunology and Genomics of Parasites of the Federal University of Minas Gerais to be processed. The eggs were isolated from the uteri of female adult worms by mechanical maceration, purified through filtration on 100 μm nylon strainers, placed in culture bottles with 50 mL of 0.2 M sulfuric acid at a concentration of 25 eggs/μL, and maintained in a BOD incubator at 26 ºC. At the 150^th^ day of culture, the peak of larvae infectivity, the fully embryonated eggs were used for the experimental infections, as we previously described [24].

### Bleomycin-induced pulmonary fibrosis mice model

For induction of pulmonary fibrosis, the mice were injected intranasally with bleomycin (Sulfate of Bleomycin, Meizle Biopharma), at a dose of 3 mg/Kg or with PBS as control, as previously described [30].

### Experimental protocol

**Fig 1:**
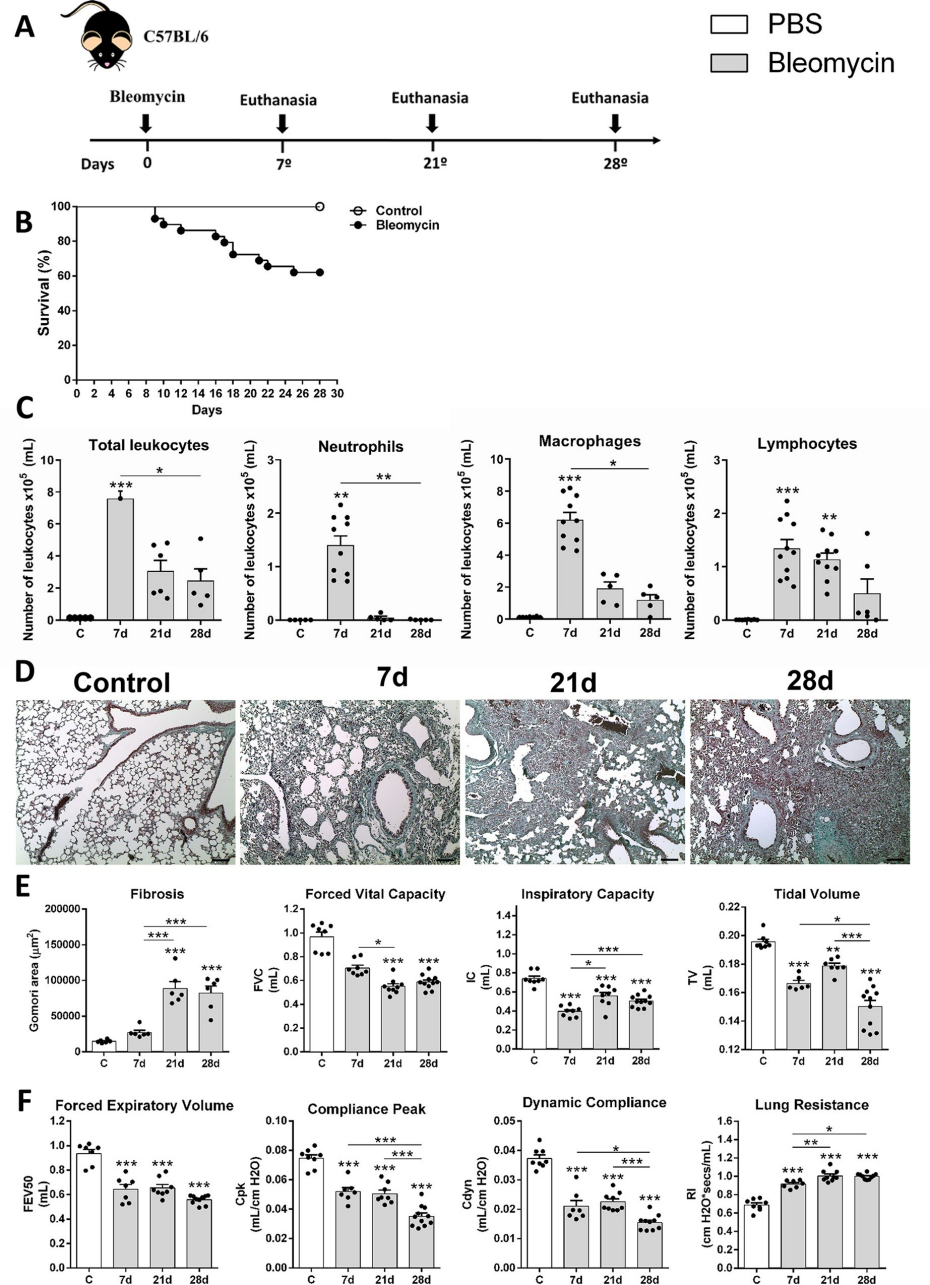
Characterization of Bleomycin-induced pulmonary inflammation and fibrosis in mice. (A) Experimental design of bleomycin-induced pulmonary fibrosis (3 mg/Kg) *in vivo* in the times 7, 21 and 28 days after instillation of bleomycin; (B) Survival curves of mice treated with saline or bleomycin-induced lung fibrosis; (C) Quantification of total leukocytes in BAL, (D) differential counts of neutrophil, macrophage, and lymphocytes infiltration in BAL; (E) Gomori’s trichrome staining of lung sections in control and necropsied mice after 7, 21, 28 days after induction of fibrosis, Bar = 100μm; (F) Morphometric analysis of the fibrosis area. Measurement of pulmonary volumes (G): FVC, IC and TV; Analysis of airway flow by Forced Expiratory Volume at 50 milliseconds (FEV50) and lung elasticity by Cpk, Cdyn and Rl (H). Results represent mean ± S.E.M., *P< 0.05, **P< 0.01, ***P< 0.001. One-way ANOVA test and Kruskal-Walis test followed by Dunn's test were used. Bar = 100μm.

**Fig 2:**
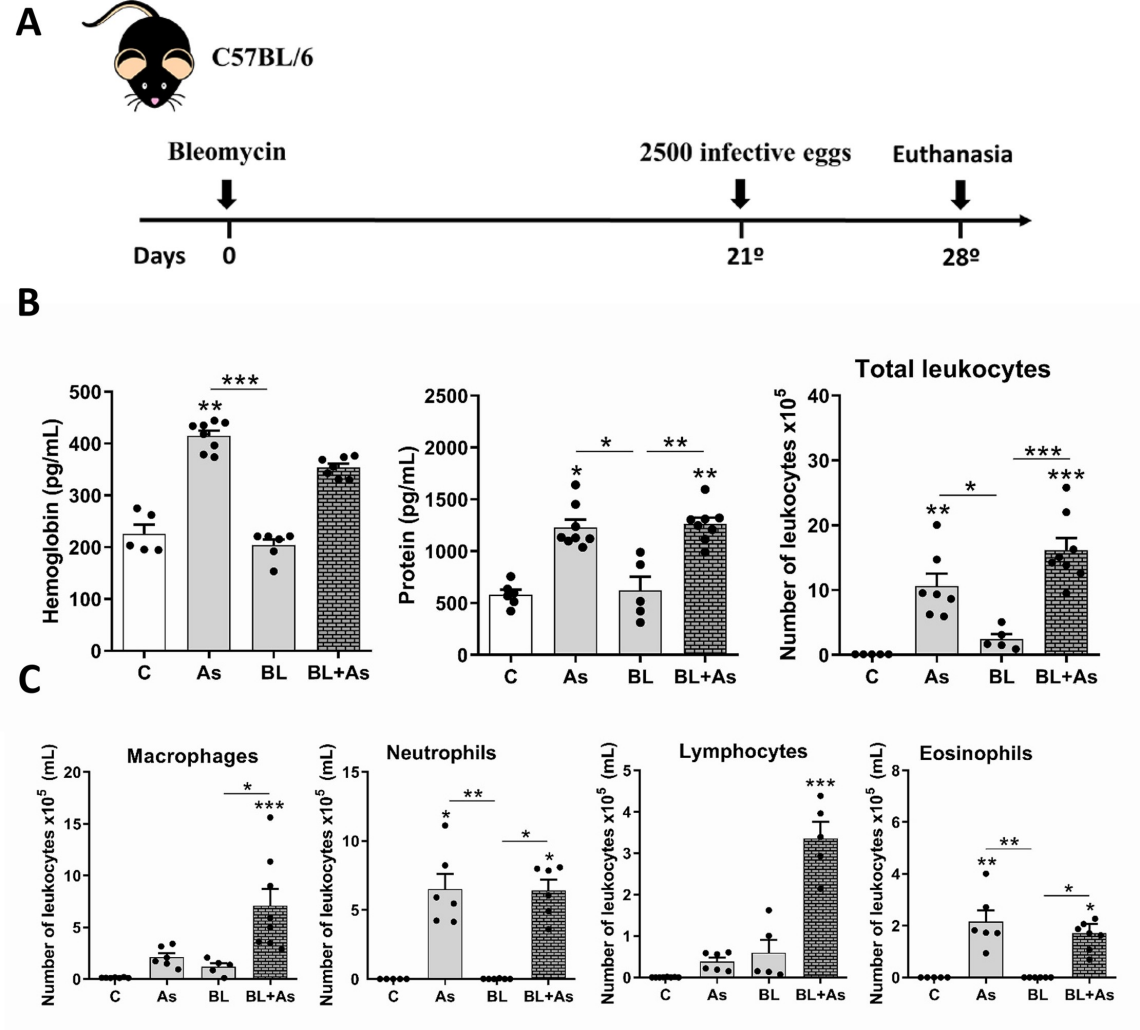
The association *Ascaris suum* with pulmonary fibrosis exacerbates airway inflammation in mice. (A) Experimental design of Comorbidity in mice, treated with saline (C group), only infected with *Ascaris suum* (As group), only bleomycin-induced lung fibrosis (BL group), and comorbidity by bleomycin-induced lung fibrosis and infected with *Ascaris suum* (BL+As group); (B) Airway inflammation: Hemoglobin, total protein levels and quantification of total leukocytes infiltration in BAL; (C) Differential quantification of leukocytes in arways, such as macrophages, neutrophils, lymphocytes and eosinophils in BAL. Results represent mean ± S.E.M., *P< 0.05, **P< 0.01, ***P< 0.001. One-way ANOVA test and Kruskal-Walis test followed by Dunn's test were used.

Mice were randomly divided into the following experimental groups: control group (C); or submitted to a single bleomycin induction, and further euthanized at 7, 21, and 28 days after instillation (BL) (Fig 1A); submitted to a single infection with *Ascaris suum*, and euthanized after 7 days (As); or submitted to a single infection of *Ascaris suum* after 21 days of fibrosis induction, and subsequently euthanized on day 28 (BL+As) (Fig 2A). Experimental designs with the experimental groups, times of infection with *Ascaris suum*, induction of bleomycin fibrosis and euthanasia are detailed in Figs 1A and 2A.

### Experimental infection and parasitological analysis

To infect mice with *A*. *suum*, a gavage needle was used, where the mice belonging to the As and BL+As groups received orally 2,500 embryonated eggs in 200 μL of PBS. The group C received only 200 μL of PBS via the same route. Parasite burden was evaluated by recovery of lungs and bronchoalveolar lavage (BAL). Tissues were collected, sliced with scissors, and placed in a Baermann apparatus for 4 h in the presence of PBS at 37°C. The recovered larvae were then fixed (1% formaldehyde in PBS), and counted under an optical microscope as previously described [24,25].

### Sample and tissue extraction

Mice were euthanized with a lethal injection of xylazine/ketamine. The bronchoalveolar lavage (BAL) was performed inserting a 1.7 mm catheter into the trachea of mice, and washing the lungs twice with 1 mL of sterile PBS three times. BAL samples (±2.7–3.0 mL each) were centrifuged at 600 × *g* for 10 min at 4°C. Supernatants were used for total protein and hemoglobin quantification, according to the manufacturer’s instructions as we previously described [24]. Subsequently, the pellet was collected, and used to evaluate the number of infiltrating leukocytes. The total number of leukocytes was determined by counting leukocytes in a modified Neubauer chamber. Differential counts were obtained from cytospin (Shandon III) preparations by evaluating the percentage of each leukocyte on a slide stained with May-Grünwald-Giemsa [7]. The right lobes of the lungs were harvested for cytokine analysis using ELISA [24]. The left lobes of the lungs and liver were prepared for histological analysis [24]. In other experiments, similarly infected animals were anesthetized with xylazine/ketamine (8.5 mg/kg and 130 mg/kg), and placed in whole-body plethysmograph for the analysis of lung mechanical changes using spirometry [30].

### Assessment of respiratory mechanic dysfunction

Pulmonary function in mice was performed as previously described [24,25,30]. Briefly, mice received a subcutaneous injection of anesthesia (to maintain spontaneous breathing), they were tracheostomized, placed in a body plethysmograph, and connected to a computer-controlled ventilator (Forced Pulmonary Maneuver System, Buxco Research Systems, Wilmington, North Carolina USA). First, an average breathing frequency of 160 breaths/min was imposed on the anesthetized animal. Under mechanical respiration the tidal volume (TV), dynamic compliance (Cdyn), and lung resistance (Rl) were determined using a resistance and compliance (RC) test. To measure the inspiratory capacity (IC) and peak of compliance (Cpk), the quasi-static *Pressure-Volume* maneuver was performed, which inflates the lungs to a standard pressure of +30 cm H_2_O, and then slowly exhales until a negative pressure of -30 cm H_2_O, thereby measuring the volume in each point of application in the lungs. Fast-flow volume maneuver was performed, and the lungs were first inflated to +30 cm H2O, and immediately after wards connected to a highly negative pressure in order to enforce expiration until -30 cm H2O. The forced vital capacity (FVC), forced expiratory volume at 50 milliseconds (FEV50), and the flow-volume curves were recorded during this maneuver. Suboptimal maneuvers were rejected, and for each test in every single mouse, at least three acceptable maneuvers were conducted to obtain a reliable mean for all numeric parameters.

### Parasitological analysis

Parasite burden from immunized and non-immunized mice was evaluated by the total number of larvae recovered from the lungs at day 7 post infection, as previously described [24,25]. Briefly, at necropsy, the lungs were collected, punctured with surgical scissors, and placed in a modified Baermann apparatus for 4 h in PBS at 37°C and 5% CO_2_. The larvae were recovered in the pellet of the apparatus, fixed in 4% formalin, and quantified by light microscopy.

### Semi-quantitative analysis

The left lobes of the lungs and liver were removed from the mice of each group. The organs were fixed in 4% formalin solution, and gradually dehydrated in ethanol to be diaphanized in xylol, and included in paraffin blocks that were cut at 4–5 microns thick and fixed on the microscopy slide [24,25]. Slides with lung and liver tissues were stained with hematoxylin and eosin for evaluation of tissue damage, and with Gomori’s trichrome stain for assessment of tissue deposition of collagen [11,25,30]. For semi-quantitative analysis of the inflammation and pulmonary fibrosis, the slides were examined under a light field optical microscope coupled to a digital image capture system (Motic 2.0). For the airway inflammation score, perivascular inflammation, parenchyma inflammation, and hemorrhage were captured; 10 random images per animal with 10× magnification, as previously described [11,24,25]. The semi-quantitative analysis of pulmonary fibrosis was performed using Ashcroft score, as previously described [8,11].

### Cytokine measurement in lungs

For evaluation of the cytokine content in lung tissue, 100 mg of lung tissue was homogenized (TissueLyser LT-Qiagen, Hilden, Germany) in 1,000 μL of phosphate buffered PBS (0.4 M NaCl and 10 mM NaPO4) supplemented with 0.05% Tween 20, 0.5% bovine serum albumin, and protease inhibitors (0.01 mM phenylmethylsulfonyl fluoride-PMSF, 0.1 mM benzethonium chloride, 10 mM of EDTA, and 20 IU of aprotinin A). The homogenates were centrifuged at 8,000 x *g* for 10 min at 4°C, and the supernatant used for cytokine quantification as we previously described [11,24,25]. Levels of IL-1β, IL-4, IL-5, IL-6, IL-12, IL-13, IL-17A, IL-33, and IFN-γ were measured using the sandwich ELISA kit (R & D Systems, USA) in accordance to the manufacturer’s instructions. The absorbance of the samples was determined using a Versa Max ELISA microplate reader (Molecular Devices, USA) at a wavelength of 492 nm.

### Evaluation of the activity of neutrophils and eosinophils in the lung tissue

The evaluation of neutrophil and eosinophil accumulation in the lungs, MPO, and EPO from tissue homogenates were measured according to a method described previously [8,11,24,25]. After tissue homogenization (TissueLyser LT-Qiagen, Hilden, Germany), the homogenate was centrifuged at 1500 × *g* for 10 min at 4°C, and the resulting pellet was examined for EPO and MPO. The extent of neutrophil accumulation in the lung tissue was quantified by assaying myeloperoxidase (MPO) activity, as previously described [8,24,25]. Eosinophil accumulation in the tissues was quantified, as previously described, by assaying eosinophil-peroxidase (EPO) activity in homogenized lung samples as previously described [11,24,25]. Results were expressed as optical densities (O.D.).

### Tissue TGF-β_1_, IL-10, and type I collagen immunostaining

To further evaluate the expression of lung TGF-β_1_, IL-10, and type I collagen, we performed immunohistochemistry as previously described [31]. Briefly, tissue sections were deparaffinized, hydrated, and washed in pH 7.2 PBS. Endogenous peroxidase activity was eliminated by treatment with H_2_O_2_ 40 v/v solution at 0.2% PBS, and unspecific binding was blocked by incubation with goat serum diluted at 1:40. Next, sections were incubated with monoclonal anti-TGF-β_1_, IL-10, and type I collagen antibody (Santa Cruz Biotechnology, Santa Cruz, USA), diluted at 1:150, 1:100, 1:50, respectively for 18 h each, then incubated with biotinylated goat anti-mouse IgG at 1:100 (Bethyl Laboratories Inc, Montgomery, USA) and peroxidase-conjugated streptavidin at 1:100 for 1 h (ZymedLaboratories Inc., San Francisco, USA). Color reaction was developed using DAB 0.05% in H_2_O_2_ 40 v/v at 0.2%. As negative control, PBS-treated sections were used (S1 Fig). All sections were stained with Harris hematoxylin, hydrated and diaphonized for Entellan (Meck Millipore) assembly.

### Quantitative analysis of the stained positive areas of TGF-β_1_, IL-10, type I collagen, and pulmonary fibrosis

The quantification of the positive areas for TGF-β_1_, IL-10, collagen type 1, and fibrosis in the lungs was performed by morphometric analysis, as previously described [7,11,31,32]. The positive areas for TGF-β_1_, IL-10, and collagen type I were selected using the pixels with brown hues, and for fibrosis we selected the area of the lung stained with Gomori’s trichrome identified by pixels with green tones, with the subsequent creation of a binary image through digital processing. The morphometric analysis was based on images from 20 randomly selected fields (total area: 56 x 10^5^ μm^2^) of the histological sections per animal. All measurements were taken with the Carl Zeiss image analyzer KS300 software (Oberkochen, Germany).

### Evaluation of enzymatic activity ALT

Alanine aminotransferase (ALT) determination was performed using a kinetic test for the evaluation of hepatocellular lesion, as previously described [33]. Briefly, the concentration of U/L of plasma alanine aminotransferase (ALT) was determined through the adaptation of the commercial kit ALT (Bioclin ref. K035, Bioclin Quibasa, Belo Horizonte, Brazil).

### Quantification of liver injury and inflammation

The quantification of the number and area of the inflammatory foci present in the hepatic parenchyma were performed using morphometric analysis as previously described [33,34]. The morphometric analysis was based on 20 images randomly captured using a digital image capture system (Motic 2.0, Hong Kong, China). Each image was evaluated with a morphometric analysis software (Motic 2.0, Hong Kong, China), where the foci of inflammatory infiltrate were delimited and counted, and the area was obtained with the aid of software tools.

### Quantitative analysis of larval fragments of *A*. *suum* present in the lung parenchyma

To evaluate whether fibrosis interferes with the migration of the *A*. *suum* larvae, slides with histological sections of the lung stained with Gomori trichrome, were visualized under a light field optical microscope coupled to a digital image capture system (Motic 2.0), and subsequently we obtained images of the entire pulmonary parenchyma of each mouse with 10X magnification. Then the captured images were visualized in the same digital image capture system (Motic 2.0) to count all the larval fragments. The observed larval fragments were grouped in the following parameters: (1) Preserved area; when found in preserved regions of the pulmonary parenchyma with absence of fibrous connective tissue, (2) Fibrosis area; when found in fibrous regions of the pulmonary parenchyma, and (3) Airways; when found in bronchi and bronchioles.

### Statistical analysis

GraphPad Prism 6 (Graphpad software, Inc., USA) was used for the statistical analysis. The Grubb's test was used to detect the outliers in the samples and non-parametric analysis of all the results were performed. To verify the distribution of the data, the D'Agostino & Pearson omnibus normality test was used. For the comparison between the parasitic burden and the number of larval fragments found in histological sections of the lung, we used the Student’s t-test. For the comparison of the production of tissue cytokines, type I collagen, total cell count, comparison of the activity of neutrophils and eosinophils, level of alanin aminotransferase, number of inflammatory foci, mean of inflammatory foci, mean of total inflammatory area, and the fibrosis score, the One-way ANOVA test was used. Kruskal-Walis test followed by Dunn's test were used for comparison of pulmonary mechanics, differential cell count, hemoglobin and protein levels, and analysis of the fibrosis area. The Chi square test was used for the survival and Flow–volume curves. All tests were considered significant when the p value ≤ 0.05. The number of mice used per group varied according to each experiment and it was obtained from statistical sample calculation (StatMate, GraphPad Inc, USA). Sample calculation was performed based on previous published data from the group.

## Results

### Characterization of bleomycin-induced pulmonary inflammation and fibrosis in mice

Firstly, we characterized the bleomycin-induced pulmonary inflammation and fibrosis in mice at the dose of 3 mg/Kg per mice. To further investigate the comorbidity in the context of *A*. *suum* infection, they were evaluated three-time points at days 7, 21, and 28 after intranasal instillation of bleomycin (Fig 1A). Bleomycin-induced lethality started from day 8 after instillation until the final time-point at day 28, with approximately 40% succumbing during pathology progression (Fig 1B). Bleomycin-induced lethality elevated the airway leukocyte recruitment at day 7 post-instillation characterizing an acute inflammatory phase, when compared with chronic fibrogenic phase at days 21 and 28, which showed decreased total number of leukocytes in the airways (Fig 1C). The leukocyte content at the inflammatory phase on day 7, was mostly characterized by a mixed leukocyte population, and a marked neutrophil and macrophages/monocytes/dendritic cells influx, which decreased along the kinetic (Fig 1C). However, the fibrogenic phase was predominantly composed of macrophages and lymphocytes without neutrophils (Fig 1C). This (Fig 1D) depicted the progression of pulmonary fibrosis induced by bleomycin as shown by Gomori’s trichrome staining, which showed a diffuse and dense area at days 21 and 28, also confirmed through morphometry of the collagen area (Fig 1E). The analysis of pulmonary mechanics was performed by forced spirometry. Despite the mechanical dysfunction presented in mice instilled with bleomycin, both inflammatory and fibrogenic phases lead to dysfunction, leading to a progressive decrease in volumes FVC, IC, and TV (Fig 1E). It also resulted in loss of airway flow as depicted by FEV50 and elasticity by reducing compliance Cpk and Cdyn, and increased lung resistance (Fig 1F).

### Comorbidity associated with *A*. *suum* infection during pulmonary fibrosis exacerbates the airway inflammation in mice

To further evaluate the impact of *A*. *suum* infection during pulmonary fibrosis, 2500 infective eggs were orally injected by gavage at day 21 after bleomycin instillation or in respective counterparts (Fig 2A). The mice submitted to infection with *A*. *suum* showed significant increased levels of hemoglobin (hemorrhage marker), protein (exudation, lung permeability), and leukocytes (inflammation) in the BAL, when compared to those in the control mice or mice submitted to pulmonary fibrosis (Fig 2B). Similarly, the results regarding hemorrhage, protein levels, and leukocytes increased in mice belonging to the comorbidity of *A*. *suum* infection group during pulmonary fibrosis (Fig 2B). Moreover, *A*. *suum* infection during pulmonary fibrosis induced a massive macrophage/monocyte/dendritic cell DC and lymphocyte influx, with the presence of granulocytes, neutrophils, and eosinophils, which were generally not found in the fibrotic phase (Fig 2C). Collectively, the results clearly indicate that *A*. *suum* infection increased inflammatory signs, such as hemorrhage, exudation, and inflammation during fibrosis, mostly due to larval migration in comorbidity.

### Comorbidity associated with *A*. *suum* infection during pulmonary fibrosis exacerbates tissue damage and chronic granulocyte tissue accumulation, but not fibrosis in mice

**Fig 3:**
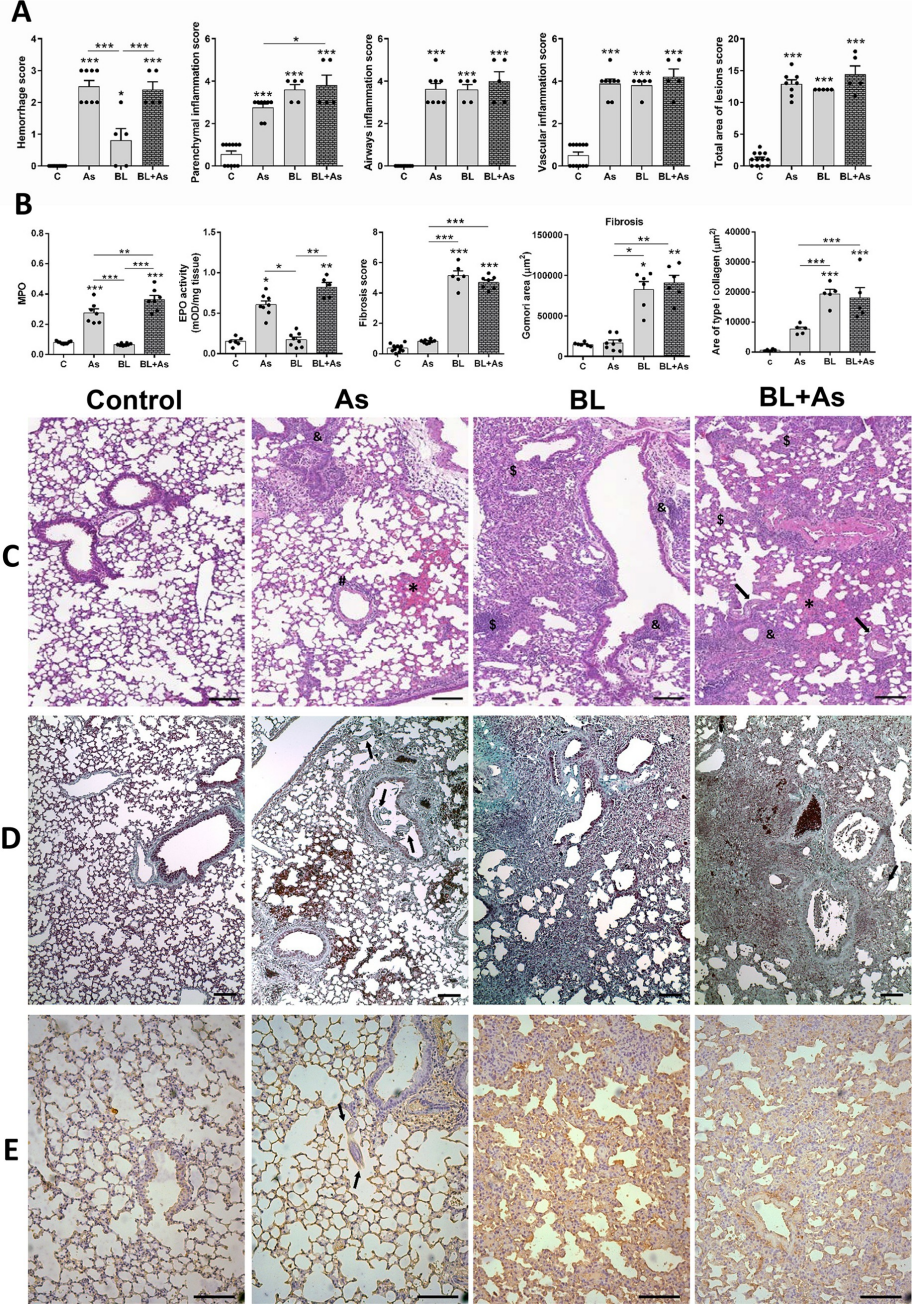
Comorbidity associated to *Ascaris suum* infection during pulmonary fibrosis increases lung parenchymal damage, but not fibrosis in mice. (A) Histopathologic score of lung injury and inflammation including Hemorrhage, parenchyma inflammation, airways inflammation, vascular inflammation, total area of lesions; (B) MPO and EPO activity in lung tissue, Ashcroft Fibrosis score (B), morphometry of the area of Gomoris thrichrome staining and morphometry of ​​ type I collagen in the lungs (B). (C) Representative hematoxylin and eosin staining of lung sections, hemorrhage area (*), parenchyma inflammation ($), airways inflammation (&), vascular inflammation (#). (D) Gomori trichrome staining of lung sections, and (E) the immunohistochemistry for type I collagen. Results represent mean ± S.E.M., *P< 0.05, **P< 0.01, ***P< 0.001. *Ascaris suum* larvae depicted by arrows, Bar = 100μm.

Histopathological analysis of the lung parenchyma allowed us to observe and describe pulmonary parenchymal lesions caused by *A*. *suum* infection and/or induced by bleomycin. Using Hematoxylin & Eosin staining, we evaluated the injury and inflammation in tissues using a score of lung injury and inflammation, as we previously described [11]. *A*. *suum* infection presented multifocal increased hemorrhagic areas with marked parenchymal, vascular, and airway inflammation, a typical damage induced by larval migration (Fig 3A). Bleomycin-induced pulmonary fibrosis displayed a more accentuated and diffuse pattern of lung injury and inflammation, however, with reduced hemorrhage compared to *A*. *suum* infection (Fig 3A). Regardless, when combined, pulmonary fibrosis plus *A*. *suum* infection mice exhibited intense and diffuse hemorrhage, caused by larval migration, and characterized by increased parenchymal inflammation compared with that of *A*. *suum* infection mice (Fig 3A).

The lung inflammation and accumulation of granulocytes during *A*. *suum* infection is a hallmark of tissue inflammation [24], however, the chronic phase of pulmonary fibrosis is more predominant with influx of macrophages [7,8]. Corroborating our previous observations, we found increased MPO and EPO activity in *A*. *suum* infected mice but not in bleomycin-induced pulmonary fibrosis (Fig 3B). The presence of exudative phenomena such as perivascular edema and exuberant hemorrhage in parenchyma areas, with peribronchial inflammation predominantly characterized by eosinophilic and neutrophilic content were observed evaluating the pulmonary tissue from *A*. *suum* infected mice (Fig 3C). However, bleomycin-induced pulmonary fibrosis exhibited diffuse microscopic lesions in lung parenchyma characterized by large thickening of the interalveolar septa and intense fibrosis, and with evident reduction of pulmonary airway spaces. The presence of perivascular edema and inflammatory infiltrate composed of predominantly lymphocytes and macrophages were observed (Fig 3C). However, during the comorbidity of *A*. *suum* infection with pulmonary fibrosis in mice, we found a more pronounced MPO and EPO activity in the lungs than in the case of *A*. *suum* infection or bleomycin-induced pulmonary fibrosis only (Fig 3B). Histopathological analysis of the lung parenchyma from mice with comorbidity allowed us to identify the presence of diffuse microscopic lesions characterized by thickening of the interalveolar septum, thickening of the septa, and the presence of extensive fibrosis, and collapse of airway spaces (Fig 3C). Exuberant hemorrhagic zones and perivascular edema and vessels with moderate thickening of the vascular wall were also observed during comorbidity (Fig 3C). The peribronchial and perivascular inflammation was composed of a mixed inflammatory cell infiltrate, predominantly eosinophils and neutrophils, and diffuse presence of lymphocytes and macrophages. We also found that in the most fibrous regions the inflammatory infiltrate was composed of lymphocytes and macrophages, however, the neutrophils and eosinophils were mostly related to injury and hemorrhage areas induced by larval migration (Fig 3C).

The multiple exposures to *A*. *suum* infection in mice led to chronic lung injury associated with tissue remodeling with modification in pulmonary extracellular matrix components as result of multiple tissue injury and healing, but not the single infection [24]. In this context, we also evaluated if the comorbidity of *A*. *suum* infection during pulmonary fibrosis could promote an excessive collagen deposition in mice. *A*. *suum* infection *per se* led to discrete fibrosis as quantified by the Ashcroft score of pulmonary fibrosis and morphometry of Gomori’s trichrome-stained area (Fig 3B), which was mostly in the perivascular and peribronchial spaces with the presence of larvae close to or occupying the lumen of the bronchi and bronchioles (arrows), as depicted in Fig 3D. Bleomycin-instilled mice showed a significantly increased pulmonary fibrosis (Fig 3B), with diffuse and dense alveolar wall thickening and collapse; also with hypertrophy and hyperplasia of the bronchial epithelial cells (Fig 3D). However, when both pathologies are combined, *A*. *suum* infection did not enhance the bleomycin-induced pulmonary fibrosis, already established at day 21 (Fig 3B and 3D). Nevertheless, it was also possible to observe the presence of larvae occupying the lumen of the bronchi and bronchioles (Fig 3D arrows). Hypertrophy and hyperplasia of the bronchial and bronchial epithelial cells were frequently observed as well as the presence of leukocytes blocking the lumen (Fig 3D). Finally, using immunostaining for type-1 collagen and morphometry, we confirmed that *A*. *suum* infection induced type-1 collagen expression predominantly in perivascular and peribronchial spaces (Fig 3B and 3E). The presence of larvae in non-fibrous areas of the parenchyma indicated that they might have sought areas free of fibrosis to migrate (Fig 3E, arrows). Contrarily, bleomycin-induced fibrosis expressed a diffuse and intense parenchymal type-1 collagen staining (Fig 3B and 3E). Corroborating our results using Gomori’s trichrome staining, the comorbidity showed similar parenchymal type-1 collagen expression in the perivascular, peribronchial spaces, and alveolar wall (Fig 3B and 3E). Collectively, our data indicates that the injury caused by comorbidity contributed to this significant increase in hemorrhage and increased neutrophil and eosinophil activity, but not fibrosis in mice.

### Characterization of cytokines related to *A*. *suum* infection and pulmonary fibrosis in mice

**Fig 4:**
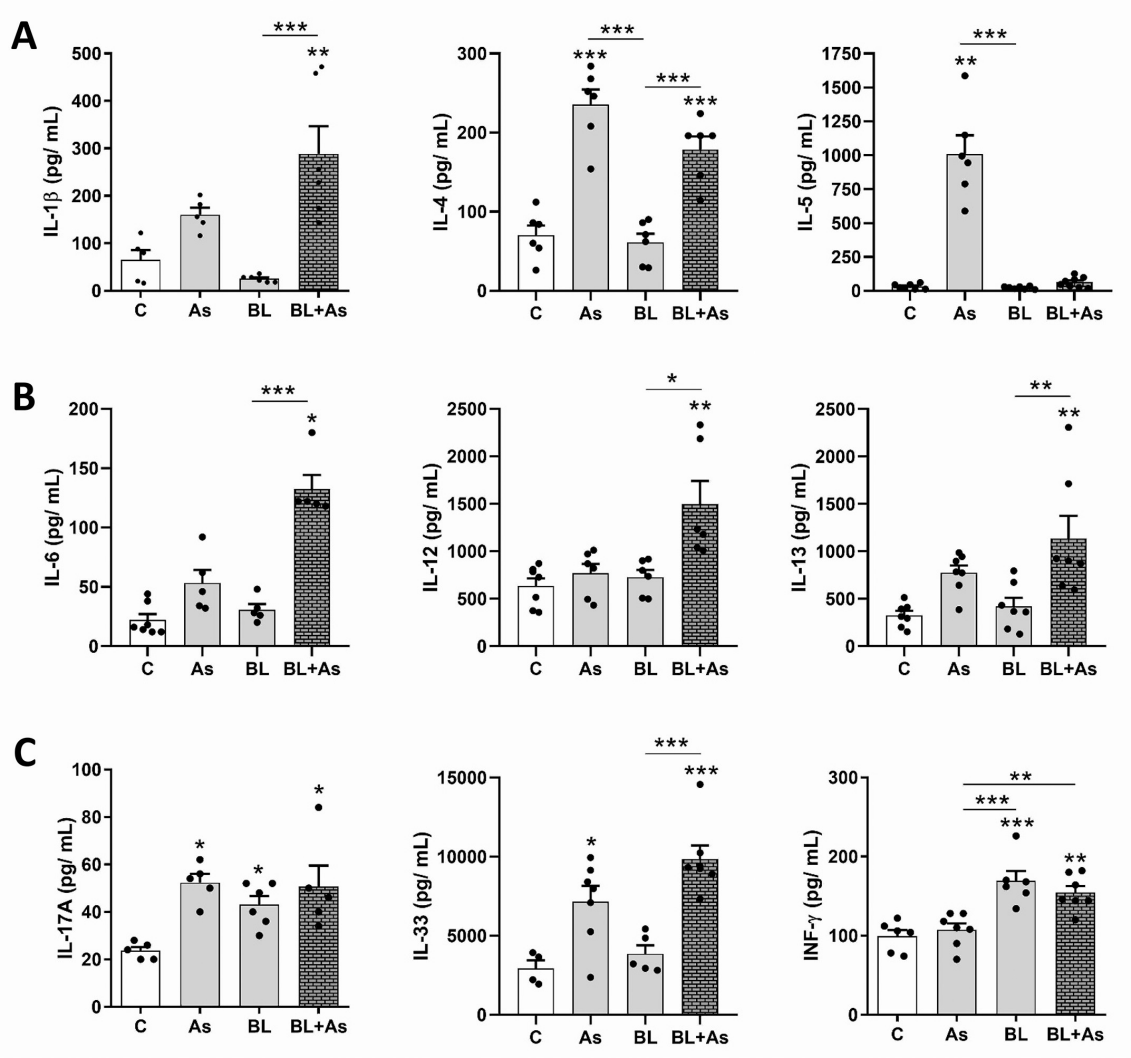
ELISA characterization of the profile of lung cytokines related to *Ascaris suum* infection, pulmonary fibrosis and comorbidity in mice. (A) Quantification of the IL-1β, IL-4, IL-5 by ELISA, (B) Quantification of the IL-6, IL-12, IL-13. (C) Quantification of the IL-17A, IL-33 and IFN-γ. Results represent mean ± S.E.M., *P< 0.05, **P< 0.01, ***P< 0.001, or One-way ANOVA test was used.

**Fig 5:**
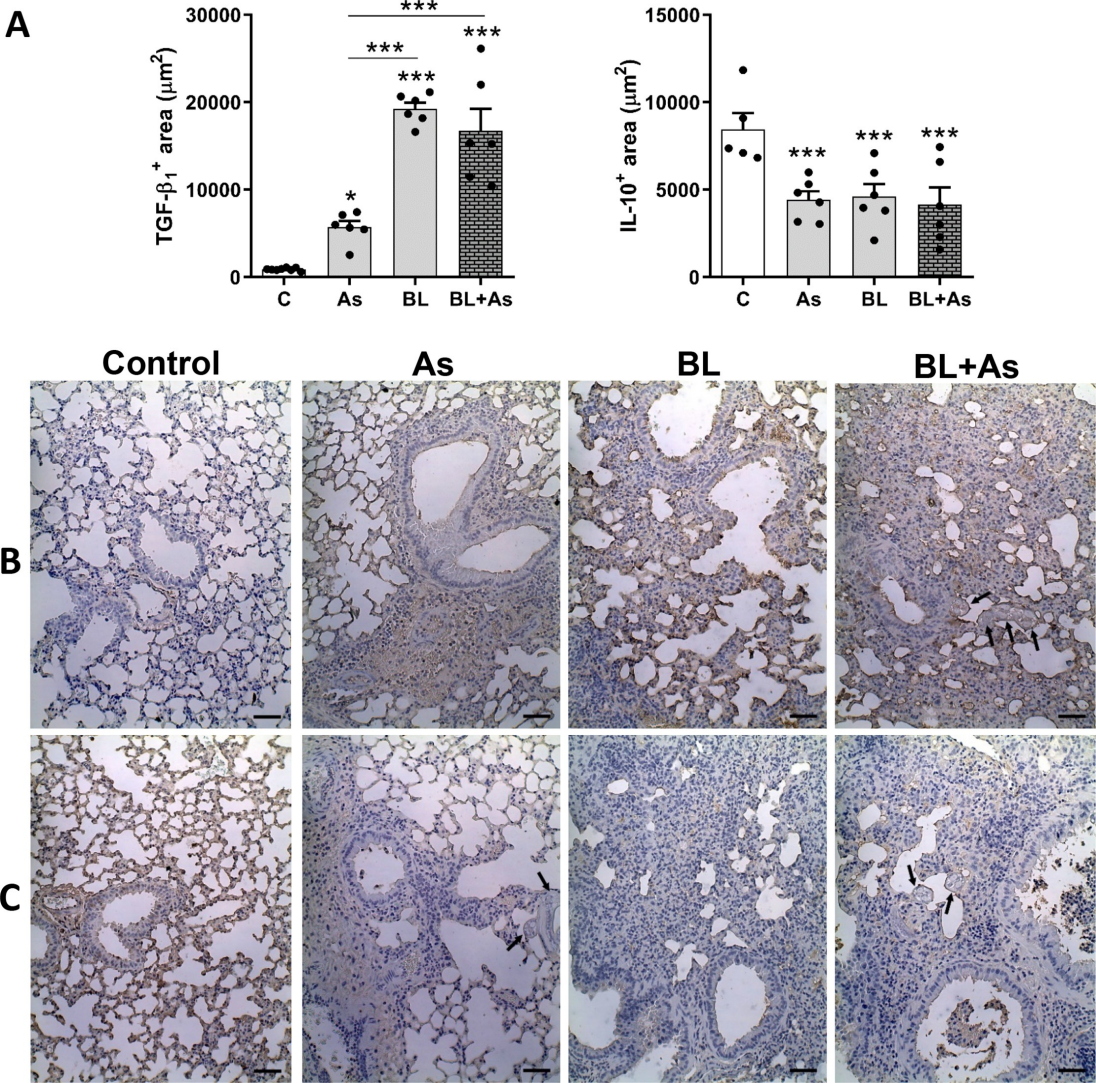
Immunohistochemical characterization of TGF-β1 and IL-10 expression in the lung parenchyma related to *Ascaris suum* infection, pulmonary fibrosis and comorbidity in mice. (A) morphometry of TGF-β1 expression in the lungs and morphometry of the positive area of IL-10 expression in the lungs. (B) Representative immunohistochemestry for TGF-β1 expression in the lungs. (C) Representative Immunohistochemistry for IL-10 in the lungs, Results represent mean ± S.E.M., *P< 0.05, **P< 0.01, ***P< 0.001, or One-way ANOVA test was used. *Ascaris suum* larvae depicted by arrows, Bar = 100μm.

Once accepted that *A*. *suum* infection and pulmonary fibrosis displayed a common Th2/Th17 cytokine pathway that determines the pathology progression [1,6,24,28], we verified whether there is some interference during comorbidity, in specific pathways of immune response involving the production of IL-1β, IL-4, IL-5, IL-6, IL-10, IL-12, IL-13, IL-17A, IL-33, TGF-β_1,_ and IFN-γ (Figs 4 and 5). Regarding IL-1β, evaluated by ELISA, and there was an increase only in the group submitted to comorbidity (Fig 4A). IL-1β is a cytokine that has been described as profibrogenic since its blockade reduced the inflammatory and resultant fibrotic response following bleomycin administration [35]. In evaluating the production of IL-4, which is a cytokine already characterized in Th2 immune response present in helminthic infections [24,28] and pulmonary fibrosis [1,6,36], we observed a significant increase in IL-4 in the *A*. *suum* infection and comorbidity groups when related to the control groups and only submitted to fibrosis induction (Fig 4A). Elevated levels of IL-5 are related to the recruitment of eosinophils, neutrophils, and cells effective against helminth infections [28]. Our results demonstrate that *A*. *suum* infection induced high levels of IL-5, on the other hand, the same was not observed in the other groups, reinforcing that the induction of fibrosis by bleomycin and consequently the comorbid condition did not alter the immune response against *A*. *suum* (Fig 4A). When IL-6 production was evaluated, the comorbidity condition contributed to a significant increase in this cytokine. The same was not observed in the *Ascaris suum* infection groups, which only submitted to fibrosis (Fig 4B). A previous study using IL-6^-/-^ mice showed deficiency in the development of a profibrogenic response, thereby highlighting the importance of IL-6 in bleomycin-induced pulmonary fibrosis [37]. In our analysis, as found with cytokines IL-1 and IL-6, there was a significant increase in IL-12 and IL-13 at the tissue levels only in the comorbid condition (Fig 4B). Elevated levels of IL-13 characterizing Th2 response have already been related to profibrogenic condition [1,6], on the other hand regarding IL-12, there is no data in the literature describing its profibrogenic role nor its relationship with *A*. *suum* infection. Although high levels of IL-12 are related to intestinal mucosal damage in *Schistosoma mansoni* infections [38], there is no related data regarding its role in *A*. *suum* infection. In our study, we did not observe significant differences in IL-12 at the tissue levels present in the *A*. *suum* infection group, reinforcing that only the comorbid condition was able to raise IL-12 levels (Fig 4B).

Regarding IL-17A, evaluated by ELISA, it was increased in all groups acutely after *A*. *suum* infection (7 d), as well as in chronic pulmonary fibrosis (28 d). Although comorbidity showed a significant increase in mice compared to *A*. *suum* infection or bleomycin-induced chronic pulmonary fibrosis, no significant differences were observed (Fig 4C).

IL-33 is a cytokine that targets immune response to Th2 following helminth infection [39], and its profibrogenic effect on bleomycin fibrosis induction has been demonstrated, mainly due to its role in M2 macrophage polarization, and expressing arginase [40]. When we performed a tissue analysis of IL-33 mice production with comorbidity, it also showed a significant increase in relation to bleomycin-induced pulmonary fibrosis. Only mice submitted to *A*. *suum* infection also showed significant increase compared to the control group (Fig 4C). Another significant profibrogenic cytokine, TGF-β_1_ [10], was evaluated by immunohistochemistry (Fig 5A and 5B). *A*. *suum* infection induced moderate levels of TGF-β_1_, and predominantly in perivascular and peribronchial spaces (Fig 5B). However, bleomycin-induced pulmonary fibrosis induced massive and diffuse expression of TGF-β_1_ in lung parenchyma, more than observed in *A*. *suum* infected mice (Fig 5B). When we evaluated the production of TGF-β1 from mice with comorbidity, it also showed a significant increase in relation to *A*. *suum* infected mice, but showed same levels as that in only bleomycin-induced pulmonary fibrosis (Fig 5A and 5B).

Cytokines like IFN-γ and IL-10 are generally related to counterbalance the progression of pulmonary fibrosis [6,41,42,43]. IFN-γ is a Th1 type cytokine and was evaluated by ELISA. Increase in the levels of IFN-γ was not induced during *A*. *suum* infection (type Th2/Th17), but was induced at then final phase of fibrosis that counterbalanced the chronic activation of TGF-β_1_. As we observed previously about IL-17A, mice with comorbidity also showed a significant increase in IFN-γ like *A*. *suum* infection, but was not reduced or increased in relation of bleomycin-induced pulmonary fibrosis (Fig 4C). Finally, we evaluated the levels of anti-inflammatory and antifibrogenic cytokine IL-10 using immunohistochemistry [41,42]. All groups, *A*. *suum* infection, bleomycin-induced lung fibrosis, and comorbidity of both pathologies, revealed equal levels of IL-10; in all groups analyzed it was reduced when compared to control mice (Fig 5A and 5C). These results demonstrate that the comorbidity of pulmonary fibrosis with *A*. *suum* infection contributed to increase in the profile of profibrogenic cytokines, but interfered in the profile of cytokines related to helminth infection.

### Comorbidity of *A*. *suum* infection during pulmonary fibrosis intensified the respiratory dysfunction in mice

**Fig 6:**
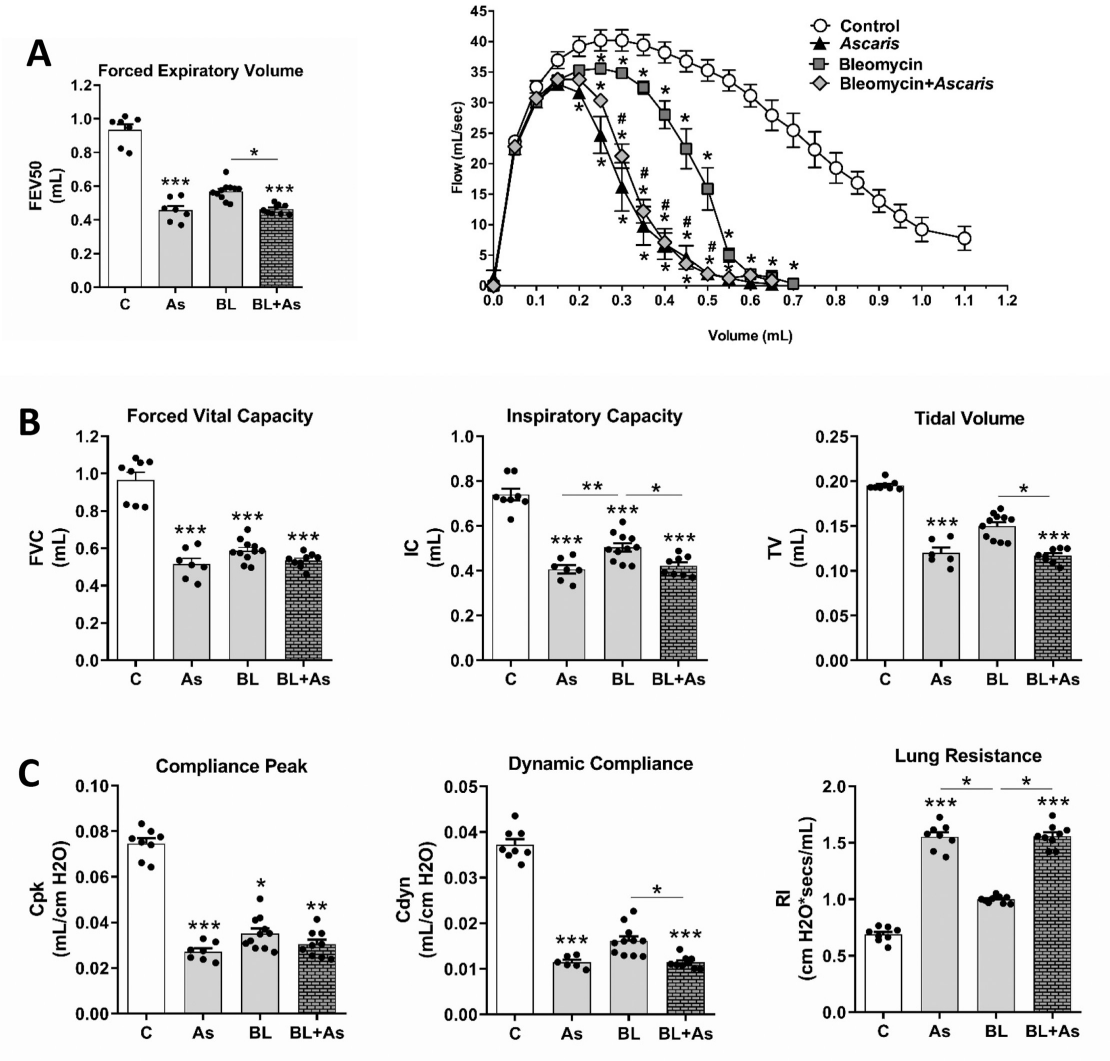
Increased respiratory dysfunction in mice with pulmonary fibrosis associated of *Ascaris suum* infection. Variables from lung mechanics were quantified as follows: Quantification of (A) Pulmonary volumes by Forced Vital Capacity, Inspiratory Capacity and Tidal Volume; Analysis of the airway flow by of Forced Expiratory Volume at 50 milliseconds (FEV50) (B) and by Flow–volume curve, (#) p <0.05 when compared to the BL group (C); and pulmonary elasticity measuring Peak of Compliance, Dynamic Compliance and Lung Resistance (D). Results represent mean ± S.E.M., *P< 0.05, **P< 0.01, ***P< 0.001. Kruskal-Walis test followed by Dunn's test were used for comparison of pulmonary Mechanics.

The analyses of pulmonary mechanics during inflammation were performed using a spirometry to investigate the physiological dysfunction caused by pulmonary pathologies. We observed that *A*. *suum* infection or Bleomycin-induced pulmonary fibrosis contributed to the loss of respiratory mechanics as observed by reduced lung volumes (Fig 6A), airway flow (Fig 6B and 6C) in which *A*. *suum* infection was more aggressive (Fig 6C), loss of pulmonary elasticity as shown by reduced compliances, and increased resistance, which was more evident in *A*. *suum* infection (Fig 6D). The comorbidity enhanced alterations in some of the variables, such as increased loss of IC and TV (Fig 6A). Moreover, the airway flow was reduced (Fig 6B and 6C), which enhanced the loss of compliance and gain in lung resistance when compared to bleomycin-induced pulmonary fibrosis in mice (Fig 6D). This result collectively demonstrates that the presence of *A*. *suum* larvae in the pulmonary parenchyma caused a significant loss of dysfunction, which was greater than bleomycin-induced pulmonary fibrosis. Notably, our data demonstrate that for most evaluated parameters the comorbidity led to greater pulmonary dysfunction when compared to the only fibrosis-induced groups.

### Comorbidity of *Ascaris suum* infection during pulmonary fibrosis promotes liver Injury and inflammation in mice

**Fig 7:**
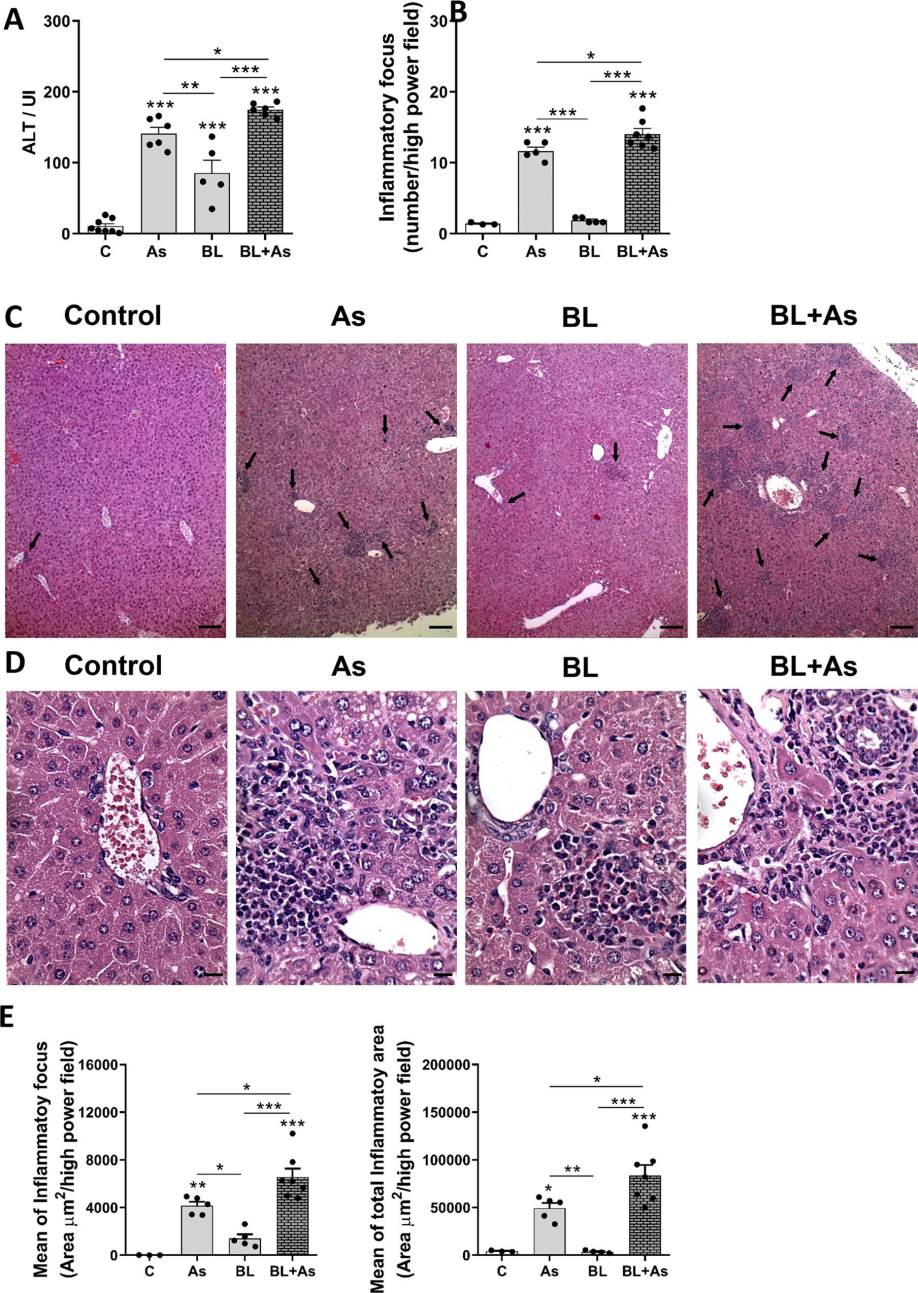
Comorbidity associated to *Ascaris suum* infection during pulmonary fibrosis promotes exacerbation of liver injury and inflammation in mice. (A) Analysis of the enzymatic activity of plasmatic alanine aminotransferase (ALT). (B) Analysis of the number of inflammatory foci found in the liver; (C) representative hematoxylin and eosin staining of liver sections, inflammatory foci (arrows); (D) representative hematoxylin and eosin staining of periportal areas with inflammatory foci (arrows) Bar = 100μm; (E) quantification of mean of inflammatory area and mean of the total inflammatory area foci present in the livers Bar = 20μm. Results represent mean ± S.E.M., *P< 0.05, **P< 0.01, ***P< 0.001. One-way ANOVA test was used.

*A*. *suum* infection leads to a progressive organ damage through mechanical injury including liver and lungs [24]. Our objective was to verify whether the proposed models *A*. *suum* infection or Bleomycin-induced pulmonary fibrosis or the comorbidity could change liver damage during the two pathologies, and having as main foci to study the damage caused in the lung. When analyzing the plasmatic levels of ALT specific marker of liver injury [33], we observed higher levels in *A*. *suum* infection but low in bleomycin-induced pulmonary fibrosis (Fig 7A). The comorbidity of *A*. *suum* infection during bleomycin-induced pulmonary fibrosis enhanced the ALT levels in lungs indicating that comorbidity contributed to increase liver damage (Fig 7A). Histopathological analyses also confirmed our previous finds. *A*. *suum* infection led to an increased number of inflammatory focus mainly caused by larval migration through the liver (Fig 7B and 7C), however, an intranasal bleomycin instillation showed low degree of inflammation (Fig 7B and 7C). Despite the comorbidity, there were increased numbers of inflammatory foci, more pronounced than that of *A*. *suum* infection only (Fig 7B and 7C).

Histopathological analysis also revealed that mice with *A*. *suum* infection displayed a moderate presence of mixed multifocal inflammatory infiltrates, characterized in large part by eosinophils, neutrophil, and to a lesser extent of lymphocytes and macrophages. The moderate presence of periportal space inflammatory infiltrates was also evident. Small areas of necrosis dispersed by the parenchyma was also observed (Fig 7D). When mice submitted to bleomycin-induced pulmonary fibrosis was evaluated, we found the presence of lesser inflammatory foci surrounding the periportal space, characterized by a leukocytes mixed population consisting of eosinophils, lymphocytes, macrophages, and lymphocytes (Fig 7D). Despite the comorbidity, we identified intense periportal space inflammatory infiltrates, with larger areas of necrosis permeated by the inflammatory infiltrates dispersed by the liver hepatic parenchyma, and mostly characterized by eosinophils, neutrophils, and to a lesser extent macrophages and lymphocytes (Fig 7D). We confirmed this inflammatory intensity by measuring the area of inflammatory foci from liver lesions. Morphometric analysis revealed that the mean and total area of inflammatory foci present in the hepatic parenchyma increased according the groups, bleomycin-induced pulmonary fibrosis, *A*. *suum* infection, and comorbidity, respectively (Fig 7E). These results demonstrate that the comorbidity association of *A*. *suum* infection during pulmonary fibrosis promotes exacerbation of liver injury and inflammation.

### Comorbidity of *A*. *suum* infection during pulmonary fibrosis did not affect mice survival and parasite burden in lungs

**Fig 8:**
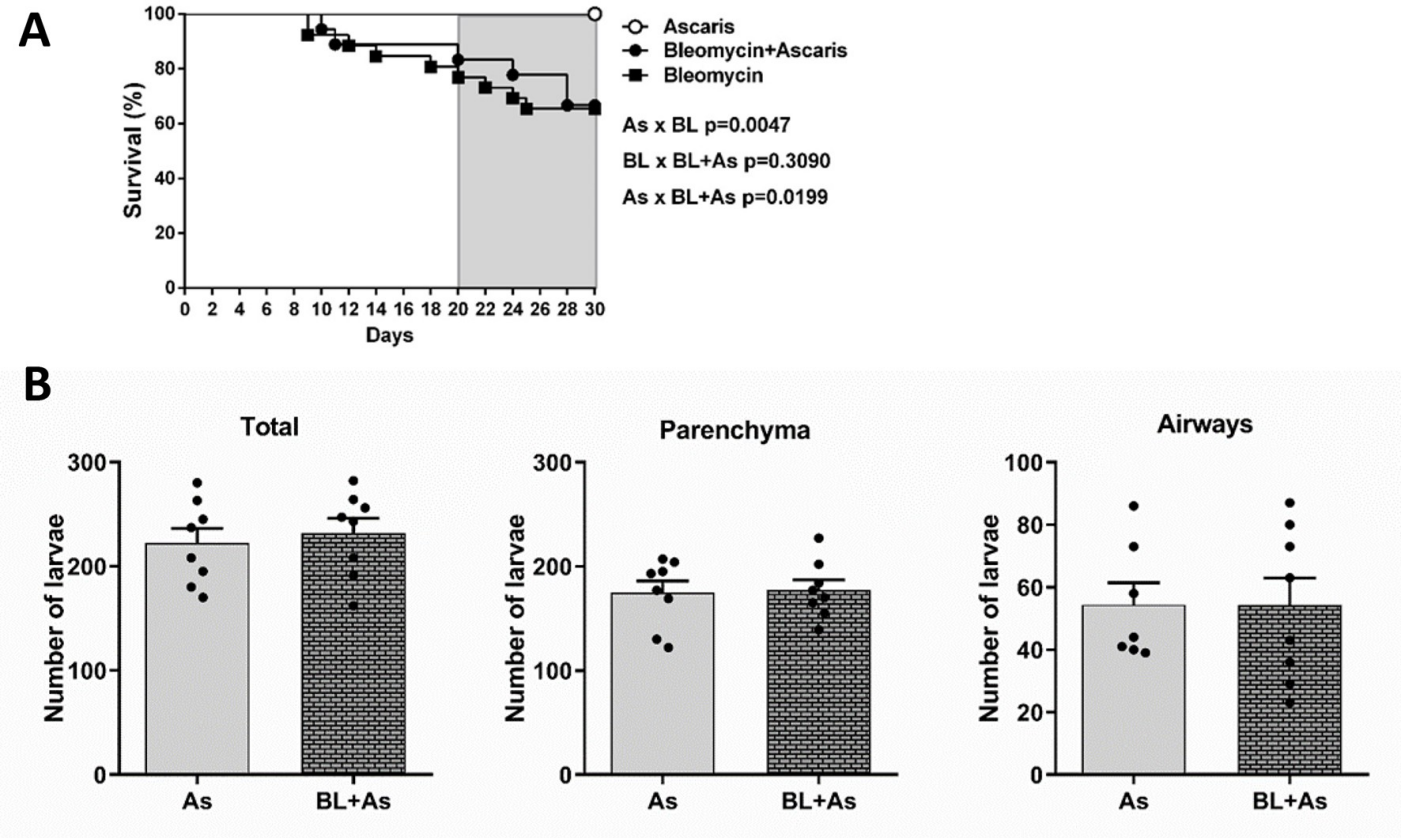
The association of *Ascaris suum* infection during pulmonary fibrosis did not impact in mice mortality and parasite burden in lungs. (A) Survival curve of mice, only infected with *Ascaris suum* (As group), only bleomycin-induced lung fibrosis (BL group), bleomycin-induced lung fibrosis and infected with *Ascaris suum* (BL+As group); (B) Total number of larvae recovered of the lungs, airways and total recovered by the bronchoalveolar lavage. Results represent mean ± S.E.M., *P< 0.05, **P< 0.01, ***P< 0.001. For the comparison between the parasitic burden and the number of larval fragments found in Berman from mice lungs, we use the test t test.

To further evaluate whether all aspects of comorbidity mentioned here could lead to increased survival, we observed mice mortality and parasite burden. We did not observe differences in mortality (Fig 8A). Next, to evaluate whether the condition of comorbidity could interfere with the parasite burden, we initially quantified the number of larvae recovered in the lungs and in bronchoalveolar lavage (BAL) from mice belonging to *A*. *suum* or comorbidity groups. All necropsies were at day 8 post-*Ascaris* eggs gavage, which corresponds to the peak of larval migration through the lungs [24]. The total number of larvae recovered from *A*. *suum* and comorbidity groups were similar (Fig 8B). We found a similar result when we compared the number of larvae recovered in the BAL *A*. *suum* and comorbidity groups that showed no significant differences (Fig 8B). In the same way, we analyzed the larvae content in the lung parenchyma, which also showed similar results in the groups *A*. *suum* and comorbidity (Fig 8B). Collectively, these results suggested that during the comorbidity, the exacerbation of lung and liver injury and inflammation did not alter the host’s survival or parasite burden in mice.

### Pulmonary fibrosis did not change the pulmonary *A*. *suum* cycle, with larvae transmigration into airways through preserved but not fibrotic areas

**Fig 9:**
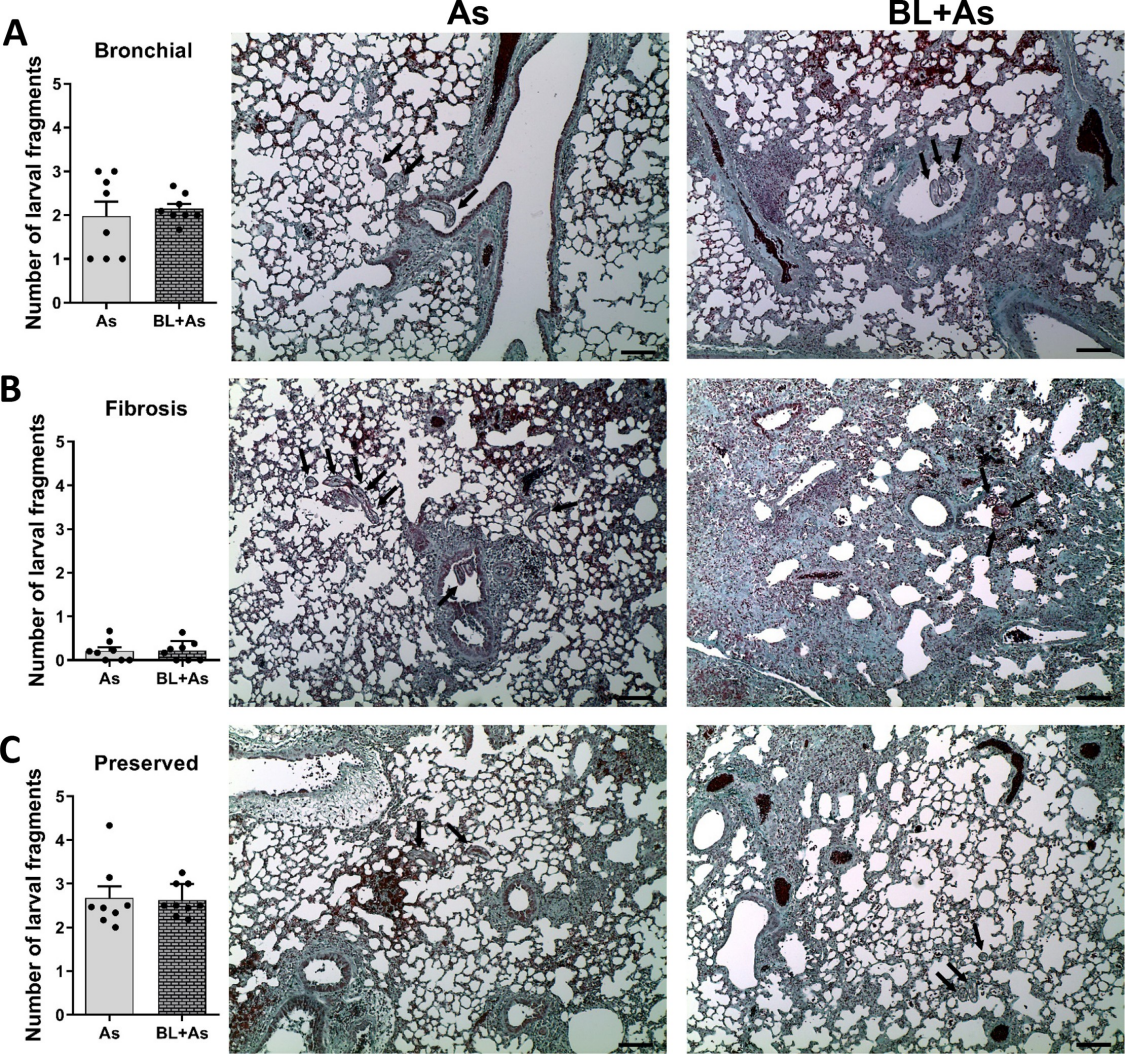
In the environment of comorbidity pulmonary fibrosis and ascaridiosis, larval migration occurs preferentially by preserved regions of the pulmonary parenchyma. Representative Gomori’s trichrome staining of lung sections and quantification: (A) Number of larval fragments quantified in the bronchial and bronchioles, (B) and representative Gomori’s trichrome staining of lung sections; (B) Number of larval fragments quantified in fibrous areas and representative gomori trichrome staining of lung sections; (C) Number of larval fragments quantified in preserved areas. We use the test t test. The results represent mean ± S.E.M. and differences between groups, are consider statistic at *P< 0.05. *Ascaris suum* larvae depicted by arrows, Bar = 100μm.

We asked whether pulmonary fibrosis formed by a dense collagen tissue could change the migration of larvae by retaining the parasite, which reflected in the number of larvae that could gain the airways to finally be ingested by host. When we confirmed that pulmonary fibrosis does not interfere in the parasitic load (Fig 8B), we proposed to investigate the behavior of *A*. *suum* larvae migration in the fibrous lung parenchyma. No significant differences were observed in the number of larval fragments present in the bronchi and bronchioles from mice infected with *A*. *suum* and comorbidity (Fig 9A). In fibrotic areas of the lung parenchyma, from mice infected with *A*. *suum* and comorbidity, few larval fragments were observed, and it was not possible to observe significant differences between the groups (Fig 9B). These results demonstrated that the fibrous pulmonary parenchyma did not interfere in the larval migration. However, a larger number of larval fragments were present in the preserved regions from both *A*. *suum* and comorbidity (Fig 9C). Moreover, when compared the counts from the number of bronchial larvae and the number of larvae related to preserved areas, they were very similar in terms of numbers (Fig 9A and 9C). Thus, our finding confirms that *A*. *suum* larvae may seek preserved areas without fibrosis to perform the transmigration and reach the bronchi and bronchioles, for further ingestion.

## Discussion

Pulmonary fibrosis is a chronic and excessive collagen deposition that results in alveolar thickening and collapsing of the tissue architecture, which affects gas diffusion, lung elasticity, and airway flow [1,2,3], and the most aggressive manifestation is the idiopathic pulmonary fibrosis (IPF) [2,3,12]. The prevalence and impact of comorbidities during the clinical course of IPF remains unclear [13] but is related to periods of exacerbations [2,3,12], including pulmonary infection, pulmonary hypertension, and lung cancer [3,12,13,14]. The *Streptococcus pneumoniae*, *Streptococcus aureus*, *Klebsiella pneumoniae*, and *Pseudomonas aeruginosa* infections are mostly common bacterial pneumonias associated with the poor prognosis and high mortality of IPF patients [14]. Based on that, the lung microbiota could contribute to the progression of IPF through lung dysbiosis, as a promoter of alveolar inflammation and aberrant repair [15]. We evaluated the role of helminthic infection by *A*. *suum* as comorbidity, because this parasite displayed an obligatory pulmonary passage to complete the cycle during chronic stages of bleomycin-induced pulmonary fibrosis in mice. In our experimental model, we used 2500 embryonated eggs to mimic the larval migration of *A*. *suum*. The need to use this inoculum was related to the characterization performed by previous studies [24,25,26], where the pulmonary alterations correlated with human parasite infection. Moreover, a significant impact on host fitness, specifically host body weight is more pronounced only at higher infection levels [44].

Considering epidemiologic data from ascariasis, it has been estimated that approximately 800 million people are infected by *Ascaris sp* per year [16,17], and recent studies showed that the prevalence of IPF was approximately 14 to 63 cases per 100,000 inhabitants per year, and most diagnoses are performed at sixth and seventh decade of life [45]. We believe that this type of comorbidity may casually happen in all continents, but is not reported or precisely diagnosable hence *Ascaris* sp infections are considered neglected disease [16,17]. This idea led us to propose two distinct hypotheses: one was about the effects of *A*. *suum* in hosts with previous lung fibrosis, and the second one was about the effects of host lung-microenvironment on the parasite cycle. We thought of two simple questions: (i) Whether *A*. *suum* infection could immuno-modulate (down-modulate) fibrogenic response or exacerbate pulmonary fibrosis leading to comorbidities, and (ii) whether the lung parenchyma fibrogenesis, which is the preferential loci of larvae influx into airways, could interfere in the pulmonary cycle of *A*. *suum* as result of increased alveolar thickening, with possible larvae retention [22] limiting the influx into airways. Our results indicated that *A*. *suum* infection led to exacerbation of pulmonary and liver injury, inflammation, and dysfunction without modification during tissue fibrosis. Moreover, we demonstrated that pulmonary fibrosis induced by bleomycin did not change the pulmonary cycle of *A*. *suum* that transmigrated into the airways preferentially through preserved but not fibrotic lung areas. In order to clarify the discussion of our data, we divided the main findings of our investigation into three topics, as follows: pulmonary inflammation and fibrosis, liver injury and inflammation, and lastly the pulmonary *A*. *suum* burden and cycle.

Inflammation is an important physiological harm of tissue defense and repair [46], in our case we have *A*. *suum* infection and pulmonary fibrosis. Generally, during tissue infection, damage, stress or malfunction, the immune response, which has a physiological purpose of maintaining the host’s defense against infections and tissue repair, is triggered; this type of tissue adaptation could generate a new pulmonary homeostasis during parasite control or tissue healing [46,47]. On the other hand, immune maladaptations recurrently from parasite infection could cause excessive inflammatory tissue damage during infection or excessive fibrosis during repair [46,47]. Our data revealed that despite pulmonary fibrosis induced by bleomycin in mice, *A*. *suum* infection exacerbated tissue damage and inflammation, with marked hemorrhage and exudation, which was getting worse by the pre-established chronic inflammatory disease fibrosis. Moreover, we found that comorbidity led to increased leukocytes, mostly macrophages/monocytes/dendritic cells, neutrophils, lymphocytes, and eosinophils. These results are consistent with the histopathological findings that demonstrated the increase hemorrhage, as well as, increased MPO and EPO activity in lungs, which were mostly related to increased neutrophils and eosinophils localized in pulmonary parenchyma. In fact, increased number of leukocytes in BAL is a worse prognosis of fibrosis. Eosinophils are involved in the inflammatory process of pulmonary fibrosis, which released other cytotoxic eosinophil products, and may contribute to the exacerbation of lung injury and fibrosis during IPF [48]. Macrophages are also important cells in the development of pulmonary fibrosis as they produce large amounts of IL-8, which is an important chemotactic pair for neutrophils, and contributes to pulmonary fibrosis by producing PDGF and TGF-β, and inducing fibroblasts and myofibroblasts to produce extracellular matrix [49]. Moreover, the BAL neutrophils predict early mortality in idiopathic pulmonary fibrosis [50] and pulmonary fibrosis correlates with the duration of tissue neutrophil activation [51]. We observed that macrophage levels remained elevated at all time points of fibrosis kinetics where comorbidity was evaluated. We hypothesized that, during the comorbidity and *A*. *suum* infection, in addition to the initial influx of monocytes and their consequent differentiation into macrophages [52], a macrophage replication might be occurring mediated by the high IL-4 levels present in the fibrosis process [36,53] and identified here in *A*. *suum* and comorbidity infection. Collectively, our data suggested that the major effect of *Ascaris suum* infection during pulmonary fibrosis was the maintenance of a chronic granulocyte inflammation and hemorrhage caused by physical damage during larval migration. Indeed, most of the pulmonary mechanical dysfunctions were detected during comorbidity, such as loss of IC, TV, FEV50, Flow–Volume curve, compliance and increased resistance were very similar to that found in *A*. *suum* infection, suggesting that inflammatory response induced by infection ascribes the worsening of pulmonary injury and dysfunction during pulmonary fibrosis induced by bleomycin in mice.

Despite the exuberant inflammatory response during comorbidity, we did not find differences between the patterns of pulmonary fibrosis, as depicted by Gomori’s trichrome and Collagen I immuno-staining in both groups: bleomycin-induced pulmonary fibrosis and comorbidity. Histopathological analysis revealed that exposure to *A*. *suum* after 8 d of infection caused large lesions in the pulmonary parenchyma, as shown by the increase in multifocal hemorrhagic areas, with edema and marked inflammation of the parenchyma, perivascular, and airway, which are directly related to the acute lesion triggered by larval migration. The lack of significant differences on pulmonary fibrosis might be related to the time of infection/induction. Possibly, a later assessment in the post-infection period might render a collagen deposition in order to repair tissue damage. In fact, previous data where a long exposition to the parasite were assessed (along with multiple parasite exposure), a significant increase in collagen deposition (that contributed to the thickening of the alveolar septum) was observed [24]. Although, we did not find differences in the production or expression of typically fibrogenic cytokines such as IL-17A and TGF-β1 [6,10,24,29] during comorbidity, it was possible to observe the tissue increase of other cytokines such as IL-1, IL-4, IL-6, IL-13, and IL-33, which are also characterized by their profibrogenic potential contributing to the development of fibrosis [6,28,29,35,36,37,40]. We emphasize that most of these cytokines, which are characterized by their fibrogenic potential, were mostly elevated only in comorbidity, except for IL-4 and IL-33, which also showed moderate levels in *A*. *suum* infection. These results demonstrate that comorbidity was able to alter the immune response, which provides a profibrogenic environment, and allows us to elucidate and assume as a shortcoming of our study that if it were maintained longer, elevated levels of IL-17A and TGF-β1 could also have been observed, as well as increased collagen deposition, thereby contributing to pulmonary fibrosis. Another interesting finding was in relation to IL-12 only in the comorbidity; it was possible to verify high levels as far as it has not been related to the profibrogenic process, and at the same time, there are no studies in the literature showing its involvement with *A*. *suum* infection. Taken together, our results demonstrate that although there were no major histopathological changes in relation to increased fibrosis, comorbidity effectively contributed to alteration of the immune response during the study period. Given these results, we can also emphasize that comorbidity did not alter the response pattern involved in *A*. *suum* infection, since cytokines such as IL-4, IL-5 and IL-33 [24,28,39], which are involved in parasite infection were also elevated in the condition of comorbidity. *A*. *suum* infection did not alter the anti-fibrogenic cytokine profile of IFN-γ [6,37], and that of the anti-inflammatory cytokine IL-10 [35,36], suggesting that *A*. *suum* infection did not induce an immunomodulatory or a protective response by cytokines, and consequently no interference occurred when associated with fibrosis. Finally, pulmonary mechanical dysfunction observed in comorbidity in relation to the *A*. *suum*-infected group might have been caused mainly by changes in the immune response, and consequent inflammatory events, but not by fibrogenic histopathological changes, which demonstrates that larval migration after 7 d of infection did not exacerbates the development of pulmonary fibrosis.

Subsequently, we proposed to evaluate the liver damage to analyze the systemic manifestations caused by *A*. *suum* infection or comorbidity, since this organ is also related to larval migration [24]. Few studies have investigated pulmonary pathologies with repercussions in other organs. The underlying mechanisms that cause extrapulmonary manifestations are not completely known. Proposed hypotheses include hypoxemia and/or hypoperfusion due to pulmonary dysfunction and circulatory depression [54] or damage mediated by a pneumonitis-induced systemic inflammatory response [55]. Furthermore, the initial effects of acid aspiration pneumonitis and its impact on the function and morphology of several organs were studied in a porcine model to verify whether hypoxemia or circulatory depression may be causal factors. Evidences showed that parenchymal involvement in hepatic and other organs, as well as histopathological findings, and neutrophil infiltration occurred in the absence of hypoxemia and circulatory instability; hence, discarding them as causal factors [56]. Here, we performed histopathological and morphometric analysis of the lesions present in the liver, which were larger and more exuberant in comorbidity, which also corroborated with high ALT level. We also demonstrated that, in addition to compromising pulmonary function, the comorbidity contributed to induce greater liver damage, marked by higher ALT levels in serum when compared to *A*. *suum* infected mice. It is also important to emphasize that mice submitted to fibrosis induction showed moderate levels of ALT in relation to the mice in the control group. ALT is a specific marker of liver damage [33], suggesting that pulmonary fibrosis induced by bleomycin could also moderate to low damage of hepatocytes. The histopathological and morphometric analysis of the lesions present in the liver also corroborated with the ALT levels. We verified that in the parenchyma of the mice in comorbidity, and found a greater number of inflammatory foci that also presented larger areas, and the presence of larger areas of necrosis were frequently observed when compared to the other groups. Collectively, we can highlight that comorbidity favored a systemic inflammation marked by liver injury and inflammation, mostly caused by larval migration and excessive leukocytes in circulation. Together the results showed that comorbidity favored not only lung injury, inflammation and dysfunction, but also promoted a remote liver injury and inflammation in response to *A*. *suum* infection combined with pulmonary fibrosis in mice. We believe that liver changes in our experimental model indicate that local inflammation in the lungs produce inflammatory mediators that can gain blood circulation, and modulate the immune response in other organs. The liver, which is an organ with high metabolism and exposed to systemic circulation acts as a metabolite filter [57], and might be more susceptible to lung inflammation.

Another central point of our study was to verify whether pulmonary tissue fibrosis could interfere in the course of the cycle of *A*. *suum* infection, since that infection depends of lung integrity and pulmonary fibrosis could delay or prevent larval migration from alveolar capillaries into the airway space by retention, a common phenomenon during *Ascaris* infection [22]. Assessing the parasite load, we quantified the number of larvae recovered in the lung parenchyma and airways from BAL, from *A*. *suum* infected and comorbidity mice. There were no differences observed in the numbers of larvae fragments recovered from the lungs or airways in mice submitted to comorbidity, when compared to mice infected only with *A*. *suum*. Therefore, these results confirm that comorbidity did not change the parasite burden and consequently did not affect the *A*. *suum* pulmonary cycle. We also checked, by identification and counting of larvae fragments, whether the larvae were present in the fibrotic areas, preserved areas, or bronchial and bronchioles lumen. Few larval fragments were present in the dense fibrotic areas from the lung parenchyma of comorbidity mice. However, when we evaluated the number of larvae fragments present in the preserved regions of the lung parenchyma, we found a larger number of larval fragments. Together these results suggested that, in relation to larval migration, the burden of *A*. *suum* larvae are not controlled by fibrogenesis from lung parenchyma, but *A*. *suum* larvae search for preserved areas without fibrosis, and they normally perform the transmigration following the sequencing of the biological cycle. Finally, this migration preferentially occurs through preserved areas, and may increase the lung injury and inflammation, thereby ascribing the pulmonary dysfunction to the comorbidity group.

In conclusion, our study described the pathophysiological manifestation of pulmonary fibrosis comorbidity associated with *A*. *suum* infection. The comorbidity contributed to worsening lung damage, due to the increased hemorrhage induced by larval migration. Additionally, the comorbidity directed the immune response to a profibrogenic profile, but did not affect collagen deposition during the study period and did not alter the response to *A*. *suum*. Moreover, the comorbidity also contributed to greater liver damage, without change in mice survival. The condition of previous fibrosis in the pulmonary parenchyma did not alter the parasitic load, did not impair the larval migration, and consequently did not alter the pulmonary *A*. *suum* cycle. The present study highlights pathological changes in lungs during comorbidities related to infectious agents, which may affect the pathology progression and morbidity. These observations indicate that parasitic transmigration through the pulmonary tissue is directly involved in the exacerbation of damage, leading to comorbidity and clinical progression. Finally, we can mention that the impact of the present study highlights the need for more exploratory research to elucidate possible pathological changes in comorbidities involving parasitic diseases, which may affect the progression of the associated morbidity. Taking into consideration the epidemiology and high rates of ascariasis in the world population, experimental models of comorbidities involving *Ascaris sp*. are assets for future research.

## Supporting information

S1 Fig Representative negative controls of lung sections staining by immunohistochemitry to identify the expression for type I collagen (N-CTRL tyoe I collagen), TGF-β_1_ (N-CTRL TGF-β_1_), IL-10 (N-CTRL IL-10). (TIF)
