# Supplementary figures and images for: Comorbidity associated to Ascaris suum infection during pulmonary fibrosis exacerbates chronic lung and liver inflammation and dysfunction but not affect the parasite cycle in mice
Source: PLoS Negl Trop Dis. 2019 Nov 25;13(11):e0007896. doi: 10.1371/journal.pntd.0007896 (PMC6901262; doi:10.1371/journal.pntd.0007896)

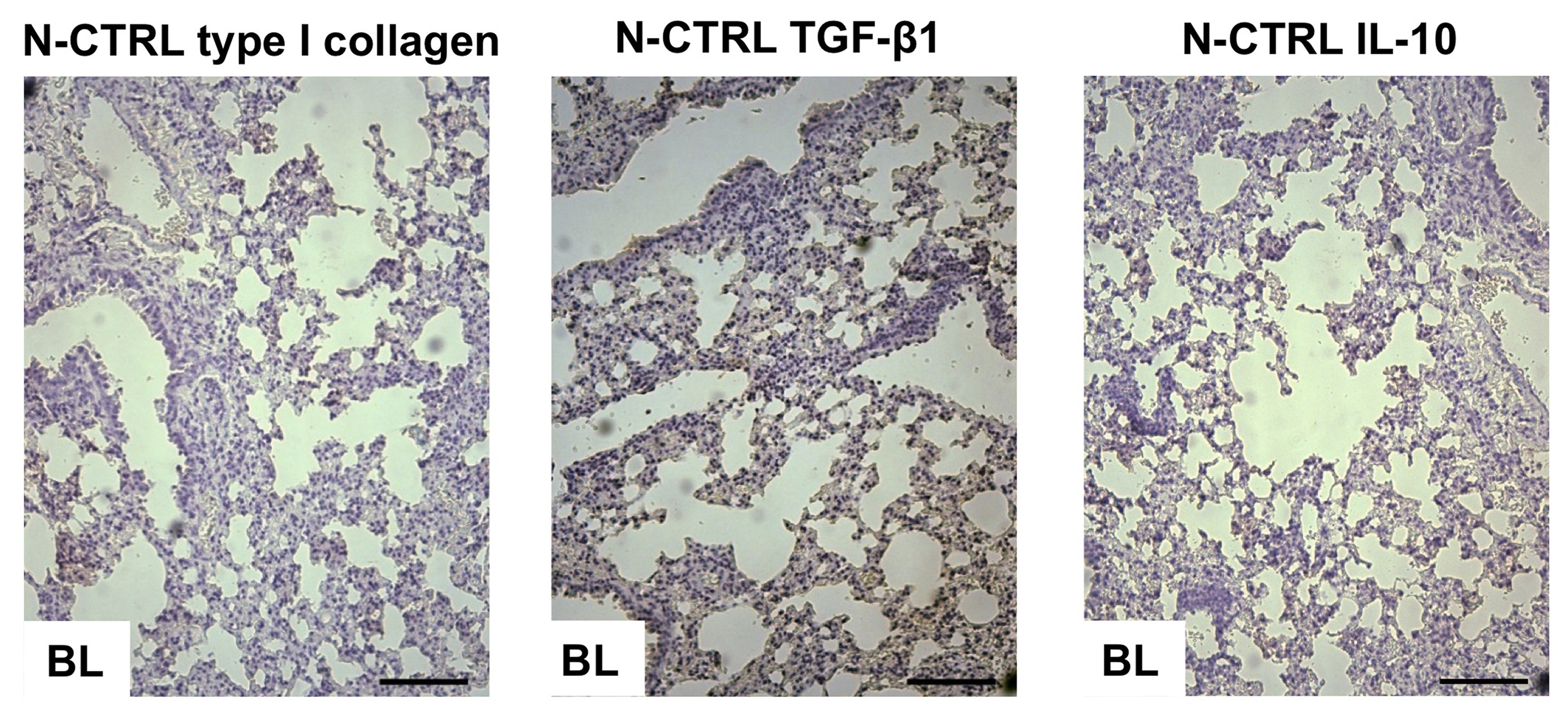

Supplement: S1 Fig — (TIF) [file pntd.0007896.s001.tif]
